# Long non-coding RNA TRPM2 antisense RNA as a potential therapeutic target promotes tumorigenesis and metastasis in esophageal cancer

**DOI:** 10.1080/21655979.2022.2033412

**Published:** 2022-02-13

**Authors:** Wei Wang, Yukai Dai, Xin Yang, Xinming Xiong

**Affiliations:** Department of Thoracic Surgery, The Second Affiliated Hospital of Guangzhou Medical University, Guangzhou China

**Keywords:** LncRNA TRPM2-AS, proliferation, metastasis, esophageal cancer, MicroRNAs

## Abstract

Esophageal cancer (EC) is one type of aggressive gastrointestinal cancers. The treatment of EC is challenging. Effective therapeutic targets require development. Long non-coding RNA TRPM2 antisense RNA (LncRNA TRPM2-AS) is considering a novel biomarker and therapeutic target for various types of cancer. However, the role of lncRNA TRPM2-AS in EC remains unknown. This study aimed to illustrate effects of LncRNA TRPM2-AS on EC growth and metastasis and potential underlying molecular mechanisms. LncRNA TRPM2-AS expression was determined in both EC tissues and cell lines by quantitative real-time polymerase-chain reaction (qRT-PCR). Cell proliferation ability was evaluated by cell counting kit-8 and colony formation assays. Cell apoptosis was analyzed by flow cytometry. Cell migration and invasion were determined using transwell. Epithelial–mesenchymal transition (EMT)-related markers expression were determined using qRT-PCR and Western blotting. Furthermore, potential lncRNA TRPM2-AS targeting miRNAs were predicted by public databases. The expression of five selected miRNAs were validated by qRT-PCR. We found that lncRNA TRPM2-AS expression was increased in EC tissues and cell lines compared with respective control. Silencing lncRNA TRPM2-AS suppressed EC cell proliferation, migration, and invasion while promoted cell apoptosis. Moreover, lncRNA TRPM2-AS knockdown reduced neural cadherin, vimentin, and matrix metallopeptidase 9 gene and protein expressions while increased epithelial cadherin expression. Furthermore, lncRNA TRPM2-AS knockdown promoted microRNA (miR)-1291, miR-6852-5p, and miR-138-5p expressions. Taken together, this study for the first time demonstrates that upregulation of lncRNA TRPM2-AS in EC promotes the growth and metastasis of EC likely through interacting with miR-1291, miR-6852-5p, and miR-138-5p.

## Graphical Abstract

**Figure:**
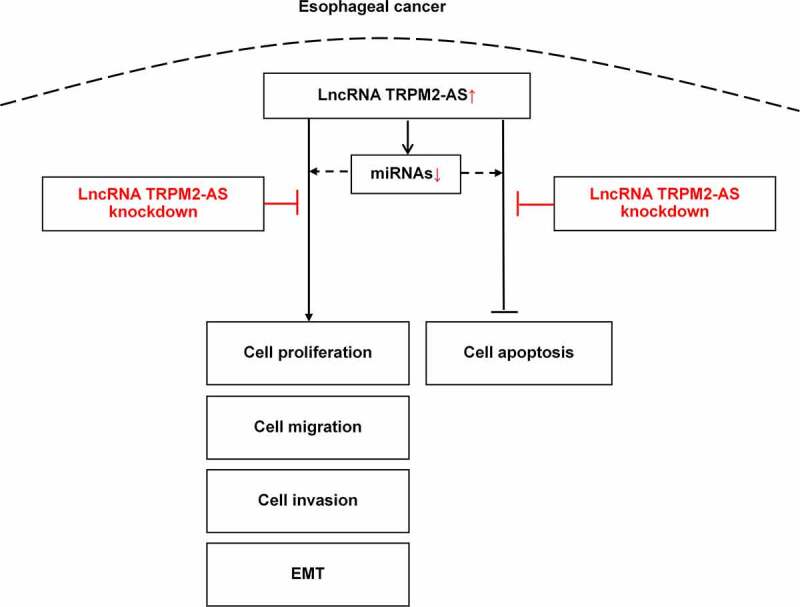

## 1. Introduction

Esophageal cancer (EC) is a gastrointestinal malignancy displaying aggressive features [1]. EC displays two major histological subtypes: esophageal squamous cell carcinoma (ESCC) and esophageal adenocarcinoma (EAC). The prevalence of EC subtypes is related to geographic regions, e.g., ESCC shows a higher incidence rate in east asia while EAC is more common in western countries [2]. In spite of a significant progress in the therapy strategies, the overall 5-year survival rate of EC patients is less than 25% [3]. The treatment of EC remains challenging and more effective drug targets are demanded developing.

Currently, a set of novel strategies applied in cancer treatment, e.g., immunotherapy, fungal-derived products, and exosomes [4–7]. There are a variety of approaches to treat EC, including surgery, radiotherapy, chemotherapy, molecular targeted therapy, and combination therapy [8]. Chemotherapy (including 5-fluorouracil, cisplatin, and doxorubicin) is still the first-line treatment for EC [8]. However, the inherent problems of chemotherapy might limit its outcomes, e.g., unspecific toxicity, drug resistance, and severe side effects [8]. Molecular targeted therapy mainly targets on epidermal growth factor receptor (EGFR), vascular endothelial growth factor (VEGF), and human epidermal growth factor receptor 2 (HER2); most of them are under clinical trials (Phase I/II) [8]. Besides, immunotherapy for EC currently has two options: immune checkpoint inhibitors and tumor vaccines, while its outcomes vary in patients due to different immune landscapes [8]. Although making significant advances in EC treatment, the development of drug resistance is a crucial factor accounting for poor outcomes. Effective therapeutic targets and strategies are urgently needed.

Long non-coding RNAs (lncRNAs) are endogenous RNAs with a length of over 200 nucleotides. It has been proved that lncRNAs play important roles in the pathogenesis of different types of tumors (e.g., ovarian cancer, clear renal cell carcinoma, and colorectal cancer) by regulating target gene expression [9–13]. Recently, accumulated evidence suggested that many lncRNAs are dysregulated in EC and regulate cell proliferation, apoptosis, and metastasis [14]. Vascular endothelial growth factor C (*VEGFC*) mRNA stability–associated long noncoding RNA (lncRNA VESTAR) promotes lymphangiogenesis and lymph node metastasis of ESCC by enhancing *VEGF-C* mRNA stability [15]. LncRNA actin gamma 1 pseudogene (AGPG) directly binds to phosphofructo-2-kinase/fructose-2,6-biphosphatase 3 (*PFKFB3*) to promote the glycolysis activity and cell proliferation in ESCC cells [16]. LncRNA cancer susceptibility candidate 9 (CASC9) was overexpressed in ESCC, promoting ESCC metastasis through upregulating laminin subunit gamma-2 (*LAMC2*) expression by interacting with the cAMP-response element-binding protein (*CREB*)-binding protein [17]. LncRNAs are providing new biomarkers and targets for the targeted therapy of EC.

LncRNA TRPM2-AS is an antisense transcript of transient receptor potential cation channel subfamily M member 2 (*TRPM2*) in the locus of chr21q22.3 [18]. Emerging evidence demonstrates that lncRNA TRPM2-AS is abnormally expressed in different types of cancers and regulates a variety of cancer cell biological process, e.g., cell proliferation, apoptosis, migration, invasion, drug resistances [18–30]. For example, lncRNA TRPM2-AS expression increased in gastric adenocarcinoma (GAC), and silencing lncRNA TRPM2-AS inhibited GAC cell proliferation and migration, while enhanced cell apoptosis [26]. A bunch of microRNAs (miRNAs) were shown to interact with lncRNA TRPM2-AS, contributing to the regulative effects of lncRNA TRPM2-AS on cell functions. In non-small cell lung cancer cells [20] and GAC cells [26], lncRNA TRPM2-AS directly bound to miR-138-5p, regulating EGFR and phosphatidyl-inositol 3-kinase/serine-threonine kinase (PI3K/AKT) signaling, and urokinase (*PLAU*) expression, respectively. LncRNA TRRPM2 also targets miR-22-3p, miR-497, and miR-612 in bladder cancer [27], retinoblastoma [23], and gastric cancer [29], respectively. Thus, lncRNA TRRPM2-AS forms a complex interaction network with miRNA regulating cancer cell biological functions. LncRNA TRPM2-AS might be a potential novel biomarker and therapeutic target for cancer treatment [31]. However, the biological functions and potential molecular mechanisms of lncRNA TRPM2-AS in EC are unknown.

We hypothesized that lncRNA TRPM2-AS might regulate EC cell biological functions by targeting downstream miRNAs. This study aimed to determine the expression of lncRNA TRPM2-AS in both EC tissues and cell lines and further evaluate the effects of lncRNA TRPM2-AS on the proliferation, apoptosis, migration, and invasion of EC cells and the potential downstream targets of lncRNA TRPM2-AS. The goal of this study is to provide potential therapeutic targets for EC treatment.

## 2. Materials and methods

### 2.1 Tissues specimen collection

A total of 15 paired EC and respective adjacent normal tissues were included in this study. These samples were collected from patients who received EC surgery in the Second Affiliated Hospital of Guangzhou Medical University during December 2019 and December 2020. The operations were approved by the Ethics Committee of the Second Affiliated Hospital of Guangzhou Medical University. Informed consents were signed by all patients. Tissues were frozen immediately and stored in liquid nitrogen after resection until subsequent experiments performing.

### 2.2 Cell culture

Human esophageal epithelial cells (HEEC) and human EC cell lines (KYSE-520 and ECA-109) were purchased from BioVector NTCC Inc. (Beijing, China). These cells were cultured with RMPI-1640 culture medium (Gibco BRL, Paisley, UK) containing 10% fetal bovine serum (FBS) in a humidified incubator (37°C, 5% CO_2_) [32]. Cells were passage when the confluence until to 80%.

### 2.3 Quantitative real-time polymerase chain reaction (qRT-PCR)

Total RNA was extracted from tissues and cells using TRizol reagent (MRC, Cincinnati, OH, USA) according to the manufacturer’s instructions. To synthesize cDNA of lncRNAs and mRNAs, 1 µg of total RNA was mixed with 1 µl random primers, and ddH_2_O was added to make the volume of 15 µl in the sterile RNase-free microcentrifuge tube. Then, the tube was heated to 70°C for 5 min to melt secondary structure within the template and subsequently was cooled immediately on ice. According to the instructions of Reverse Transcription System (Promega, Madison, WI, USA), 5 µl buffer, 6.25 µl of 2 mM dNTPs, 12.75 µl ddH_2_O, and 1 µl M-MLV were added to the tube followed by incubating at 37°C for 1 h [33]. The cDNA synthesis of miRNAs was carried out as the above procedures but used the RT primers (showed in Table 1, in which the primer with an RT suffix) of the target gene instead of random primers. PCR reaction was performed on ABI7500 system according to the instructions of ChamQ^TM^ Universal SYBR® qPCR Master Mix (Vazyme, Nanjing, China). The parameters of qRT-PCR cycling were as below: 95°C, 2 min; 40 cycles of 95°C, 15 sec; and 60°C, 30 sec. The primer sequences were showed in Table 1. *U6* was used as the internal control for miRNA expression. *GAPDH* was used as the internal control of lncRNA TRPM2-AS and mRNAs expression. The relative expression was calculated using 2^−ΔΔCT^ method.

**Table 1.** The primer sequence in the study

Primer name	Sequence (5’-3”)
TRPM2-AS:1-F	CCCGAGGAAGGCTACTGATG
TRPM2-AS:1-R	GGCTGAGTGACGAGAAGCAA
hsa-miR-138-5p-RT	GTCGTATCCAGTGCAGGGTCCGAGGTATTCGCACTGGATACGACCGGCCT
hsa-miR-138-5p-F	GCGAGCTGGTGTTGTGAATC
hsa-miR-93-3p-RT	GTCGTATCCAGTGCAGGGTCCGAGGTATTCGCACTGGATACGACCGGGAA
hsa-miR-93-3p-F	TCGTACATACTGCTGAGCTAG
hsa-miR-1291-RT	GTCGTATCCAGTGCAGGGTCCGAGGTATTCGCACTGGATACGACACTGCT
hsa-miR-1291-F	TAATTGGCCCTGACTGAAGAC
hsa-miR-6852-5p-RT	GTCGTATCCAGTGCAGGGTCCGAGGTATTCGCACTGGATACGACCATGTC
hsa-miR-6852-5p-F	TACTATCCCTGGGGTTCTGAG
Universe-R	GTGCAGGGTCCGAGGT
hsa-U6-F	CTCGCTTCGGCAGCACA
hsa-U6-R	AACGCTTCACGAATTTGCGT
hsa-miR-218-5p-RT	GTCGTATCCAGTGCAGGGTCCGAGGTATTCGCACTGGATACGACACATGG
hsa-miR-218-5p-F	GCGTTGTGCTTGATCTAA
H-vimentin-F	AACTTAGGGGCGCTCTTGTC
H-vimentin-R	TGAGGGCTCCTAGCGGTTTA
H-N-cadherin-F	GTGCATGAAGGACAGCCTCT
H-N-cadherin-R	TGGAAAGCTTCTCACGGCAT
H-MMP9-F	TGAACATCTTCGACGCCATC
H-MMP9-R	ACTTGTCGGCGATAAGGAAGG
H-E-cadherin-F	GAGAAACAGGATGGCTGAAGG
H-E-cadherin-R	TGAGGATGGTGTAAGCGATGG
H-GAPDH-F	GAGTCAACGGATTTGGTCGT
H-GAPDH-R	GACAAGCTTCCCGTTCTCAG

### 2.4 Small interfering RNAs (siRNAs) synthesized and transfection

LncRNA TRPM2-AS siRNAs (si-TRPM2-AS) and respective negative control sequences (si-NC) were synthesized by Genepharma (Shanghai, China). The sequences were as belows: si-TRPM2-AS sense: 5’-CCACCAGCCACUUACUCAU-3’, si-TRPM2-AS antisense: 5’-AUGAGUAAGUGGCUGGUGG-3’; si-NC sense: 5’-UUCUCCGAACGUGUCACGUTT-3’, si-NC antisense: 5’-ACGUGACACGUUCGGAGAATT-3’.

SiRNAs transfections were performed following the instructions of lipofectamine 3000 regent (Thermofisher, USA). In brief, KYSE-520 and ECA-109 cells were seeded into 6-well plates, respectively. When cell confluence reached 80–90%, lipofectamine 3000 and siRNAs were diluted with opti-MEM (Gibco BRL), respectively. Then, both diluted reagents were mixed and kept at room temperature for 5 min. The lipid-mixture were added on cells. Cells were cultured for additional 48 h followed by subsequent experiments, i.e., CCK-8 assay, transwell assay, flow cytometry, colony formation, and Western blotting.

### 2.5 Cell Counting Kit-8 (CCK-8) assay

After transfection, cells were digested with trypsin (Gibco BRL) to make cell suspension and seeded into 96-well plates with a density of 3000 cells in 100 μl medium. CCK-8 assay was performed following the manufacturer’s instructions (Dojindo, Tokyo, Japan). CCK-8 reagent (10 µl) was added to each well. Then cell proliferation at 24 h, 48 h, and 72 h was measured by determining the absorbance value at 450 nm using a microplate reader (Detie Lab, HBS-1096C Pro, Nanjing, China).

### 2.6 Transwell assay

After transfection as described above, KYSE-520 and ECA-109 cells were made cell suspension as CCK-8 assay did. For invasion evaluation, the transwell membrane was precoated with matrigel (BD Biosciences, #356,234, USA) at 37°C for 2 h. Cell migration assay were performed without matrigel precoating. Then, 1 × 10^6^ cells suspended in FBS-free medium were seeded into the upper chamber (BD Biosciences). The bottom chamber was added with culture medium containing 10% FBS. After incubation for 48 h, the migrated cells were stained with 1% crystal violet for 10 min followed by washing with phosphate buffer saline for one time. The migrated cells were imaged and counted under an inverted microscope (Olympus, Tokyo, Japan).

### 2.7 Flow cytometry

Cell apoptosis was determined using flow cytometry using Annexin V-APC/7-AAD apoptosis kit (MultiSciences, Hangzhou, China). According to the manufacturer’s instructions, cells were collected by centrifuging at 5, 000 × g for 5 min, washed with (1 ml) binding buffer three times, and resuspended with binding buffer to a concentration of 1 × 10^7^ cells/ml. Afterward, 100 μl cell suspension was transferred into a flow cytometry tube, mixed with Annexin V-APC (5 μl) and 7-AAD (10 μl) reagents, and incubated for 15 min at room temperature in the dark. Finally, the fluorescence signals were analyzed by flow cytometry (Beckman, Miami, FL, USA).

### 2.8 Colony formation assay

KYSE-520 and ECA-109 cells were seeded into 6-well plates with a total number of 1000 cells/well and cultured in 5% CO_2_ humidified incubator for additional 2 weeks until the cell clones were observed. Then clones were fixed with 4% paraformaldehyde and staining with 1% crystal violet. The colony images were captured under an optical microscope (Olympus).

### 2.9 Construction of lncRNA TRPM2-AS-miRNA network

The potential lncRNA TRPM2-AS targeting miRNAs were predicted using miRanda (http://www.microrna.org) and miTarbase (http://miRTarBase.cuhk.edu.cn/). Among the miRNAs list, those with top 20 scores were selected to construct lncRNA–miRNA interaction network using cytoscape (http://cytoscape.org).

### 2.10 Western blotting

Cells were lysed by RIPA buffer (Beyotime Biotechnology, Shanghai, China) supplemented with protease inhibitors (Boster, Wuhan, China) on ice for 30 min followed by centrifuging at 12, 000 × g for 30 min at 4°C. The protein concentration was determined using a BCA kit (Thermofisher) following the manufacturer’s instructions. Protein samples (25 μg) were electrophoresed with 10% sodium dodecyl sulfate, sodium salt polyacrylamide gelelectrophoresis gel, and then transferred to polyvinylidene difluoride (PVDF, Millipore, Billerica, MA, USA) membrane. The blotting membrane was blocked with 5% skim milk (Sangon biotech, Shanghai, China), and incubated with indicated primary antibodies at 4°C overnight. Then, the membrane was incubated with corresponding HRP-conjugated secondary antibodies at room temperature for 2 h. Finally, the blotting images were obtained with a chemiluminescence imaging analyzer (GE LAS‐4000, GE healthcare Life. Sciences) after incubating with enhanced chemiluminescence (ECL) reagent (Beyotime Biotechnology). The primary antibodies information were as below: anti-E-cadherin (dilution 1:1000; Cell Signaling Technology, #3195, USA), anti-N-cadherin (dilution 1:1000; Cell Signaling Technology, #13,116,), anti-vimentin (dilution 1:1000; Santa Cruz Biotechnology, #sc6260, USA), anti-MMP-9 (dilution 1:1000; Abcam, #ab38898, USA), and anti- Glyceraldehyde 3-phosphate dehydrogenase (GAPDH, dilution 1:20,000; Proteintech, #60,004-1, USA). GAPDH was used as the internal control. The secondary antibodies were as below: HRP conjugated goat anti-mouse IgG (H + L) (dilution 1:5000; Proteintech, #SA00001-1), HRP conjugated goat anti-rabbit IgG (H + L) (dilution 1:5000; Proteintech, #SA00001-2).

### 2.11 Statistical Analysis

Data were presented as Mean ± Standard deviation (SD). The Student’s t-test was used to compare the difference between means of two groups, and one-way analysis of variance was used to compare the difference between means of three or more groups. P < 0.05 was considered statistically significant. In this study, all experiments were repeated at least three times.

## 3. Results

In this study, we hypothesized that lncRNA TRPM2-AS might regulate EC cell biological functions by targeting downstream miRNAs. First, we compared the expression level of lncRNA TRPM2-AS between EC tissues and the adjacent normal tissues and further verified it between EC cell lines and HEEC. Next, we silenced lncRNA TRPM2-AS in EC cell lines and thus evaluated the effects of lncRNA TRPM2-AS on cell proliferation, apoptosis, migration, and invasion. We also conducted bioinformatic analyses to discover the potential lncRNA TRPM2 targeting miRNAs and constructed the interaction network. Finally, we used qRT-PCR to validate the regulative effects of lncRNA TRPM2-AS on the expression of several miRNAs. Our findings revealed that lncRNATRPM2-AS expression was higher in EC tissues and cell lines than that in adjacent normal tissues and HEEC, respectively. Silencing lncRNA TRPM2-AS inhibited EC cell growth and metastasis, while enhanced cell apoptosis. We further found that lncRNA TRPM2-AS might target a total of 401 miRNAs. Finally, we validated that lncRNA TRPM2-AS negatively regulated miR-1291, miR-6852-5p, and miR-138-5p. In summary, this study provides in vitro evidence that lncRNA TRPM2-AS positively regulates EC growth and metastasis likely through targeting downstream miRNAs including miR-1291, miR-6852-5p, and miR-138-5p. Our findings might provide potential therapeutic targets for EC treatment.

### 3.1 LncRNA TRPM2-AS expression is upregulated in EC tissues and cell lines

**Figure 1.**
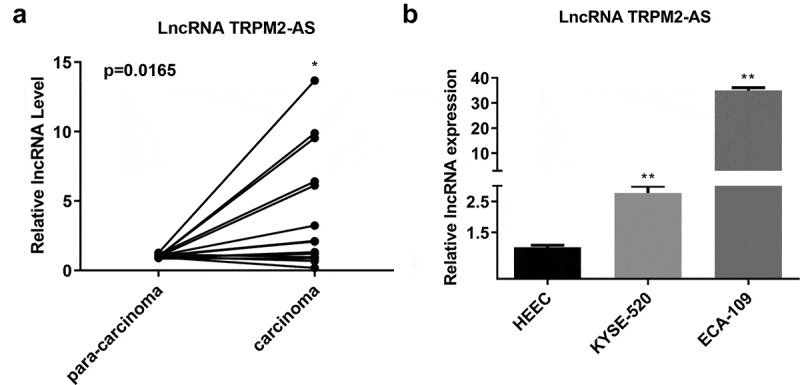
LncRNA TRPM2-AS was overexpressed in esophageal cancer (EC) tissues and cell lines.

First, we determined the expression level of lncRNA TRPM2-AS in 15 paired EC and adjacent normal tissues. As shown in (Figure 1a), lncRNA TRPM2-AS expression significantly increased in EC tissues compared with the paired pericarcinomatous tissues (p = 0.0165). LncRNA TRPM2-AS expression level was further compared between human EC cell lines (KYSE-520 and ECA-109 cells) and HEEC. The lncRNA TRPM2-AS expression levels in both KYSE-520 cells and ECA-109 cells were higher than HEEC (Figure 1b).

(A) lncRNA TRPM2-AS expression was determined in 15 paired EC and adjacent normal tissues using quantitative real-time polymerase-chain reaction (qRT-PCR). (B) LncRNA TRPM2-AS expression was determined in human EC cell lines (KYSE-520 cells and ECA-109 cells) and human normal esophageal epithelial cells (HEEC) using qRT-PCR. *p < 0.05, ** *p* < 0.01, n = 3.

### 3.2 LncTRPM2-AS silencing suppresses EC proliferation while promotes apoptosis in EC cells

**Figure 2.**
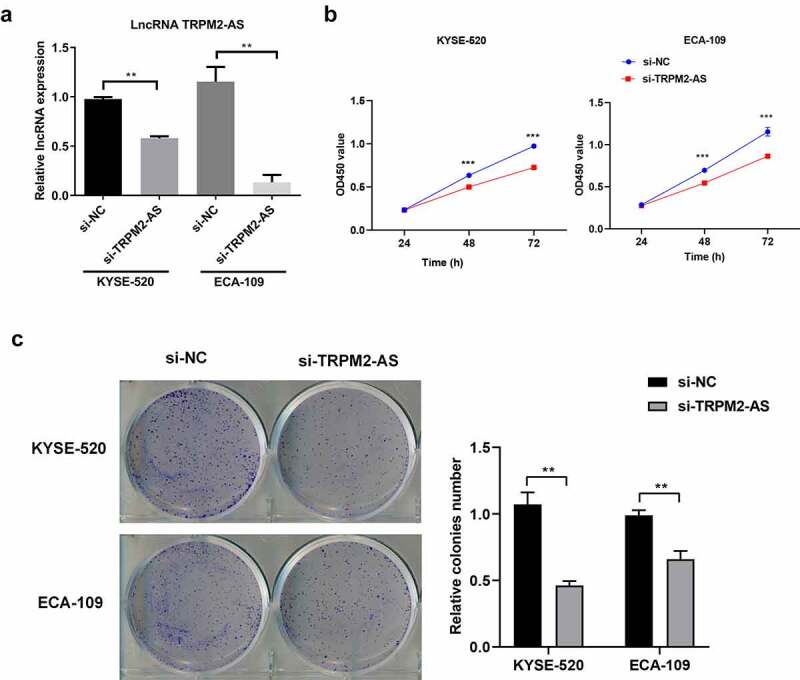
Silencing of lncRNA TRPM2-AS suppresses cell proliferation in esophageal cancer (EC) cells.

**Figure 3.**
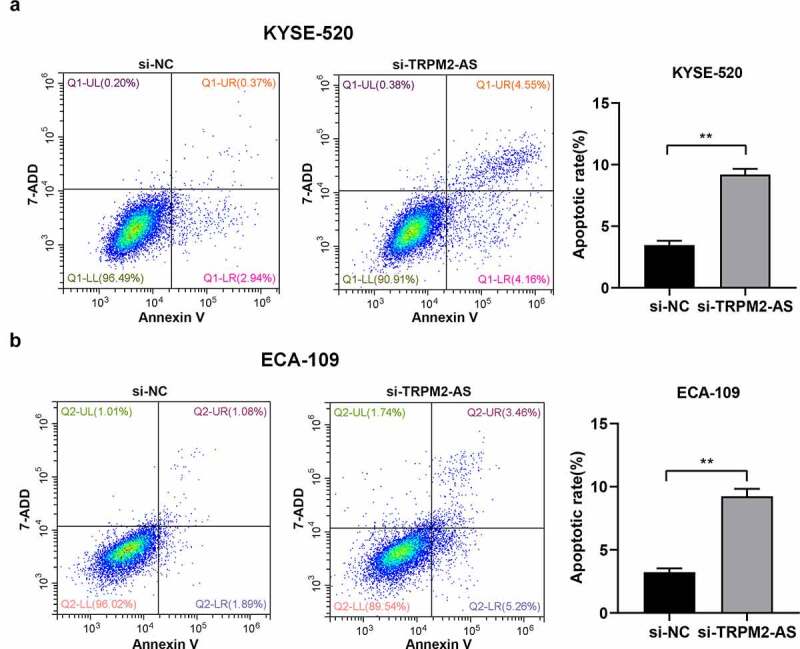
Silencing of lncRNA TRPM2-AS promotes cell apoptosis in esophageal cancer cells.

Next, we investigated the cell biological functions of lncRNA TRPM2-AS in EC. We silenced lncRNA TRPM2-AS in both EC cell lines using lncRNA TRPM2-AS specific siRNA (si-TRPM2-AS). As shown in (Figure 2a), si-TRPM2-AS reduced lncRNA TRPM2-AS expressions in KYSE-520 and ECA-109 cells by 40.6% and 88.4% compared with si-NC, respectively. Subsequently, the effects of lncRNA TRPM2-AS on EC cell proliferation were evaluated by CCK-8 and colony formation assay. We observed that lncTRPM2-AS knockdown inhibited the proliferation in both EC cell lines in a time-dependent manner (Figure 2b). LncRNA TRPM2 silencing reduced cell proliferation rates in KYSE-520 and ECA-109 cells at 48 h by 21.4% and 21.7% compared with si-NC, respectively (p < 0.05). In comparison, the inhibitive effects of lncRNA TRPM2-AS knockdown on cell proliferation at 72 h were more pronounced. Cell colony formation is used to evaluate the proliferative ability of single cells [34]. In line with CCK-8 assay results, si-TRPM2-AS significantly reduced colony formation in both EC cell lines ((Figures 2C), 62.1% of control and 66.8% of control in KYSE-520 cells and ECA-109 cells, respectively). In contrast, flow cytometry assay showed that lncRNA TRPM2-AS knockdown significantly increased apoptotic rate in KYSE-520 cells and ECA-109 cells compared with the control group (Figures 3A,B). Together, these results demonstrate that lncRNA TRPM2-AS silencing suppresses cell proliferation while enhances cell apoptosis in EC cells.

(A) The knockdown efficiency was determined by quantitative real-time polymerase-chain reaction in EC cell lines after transfecting with si-TRPM2-AS and si-NC for 48 h, respectively. (B) Cell proliferation at indicated timepoints was determined by cell counting kit-8 assay in EC cell lines after silencing of lncRNA TRPM2-AS. (C) Colony formation assay results of KYSE-520 cells and ECA-109 cells. Colony formation assays were carried out 2 weeks after silencing of lncRNA TRPM2-AS. **p < 0.01, ***p < 0.001, n = 3.

(A) Presentative images of flow cytometry assay were shown. Flow cytometry was carried out after 48 h post si-TRPM2-AS or si-NC transfection in KYSE-520 cells. (B) Flow cytometry was carried out after 48 h post si-TRPM2-AS or si-NC transfection in ECA-109 cells. ** *p* < 0.01, n = 3.

### 3.3 LncRNA TRPM2-AS knockdown inhibits cell migration and invasion in EC cells

**Figure 4.**
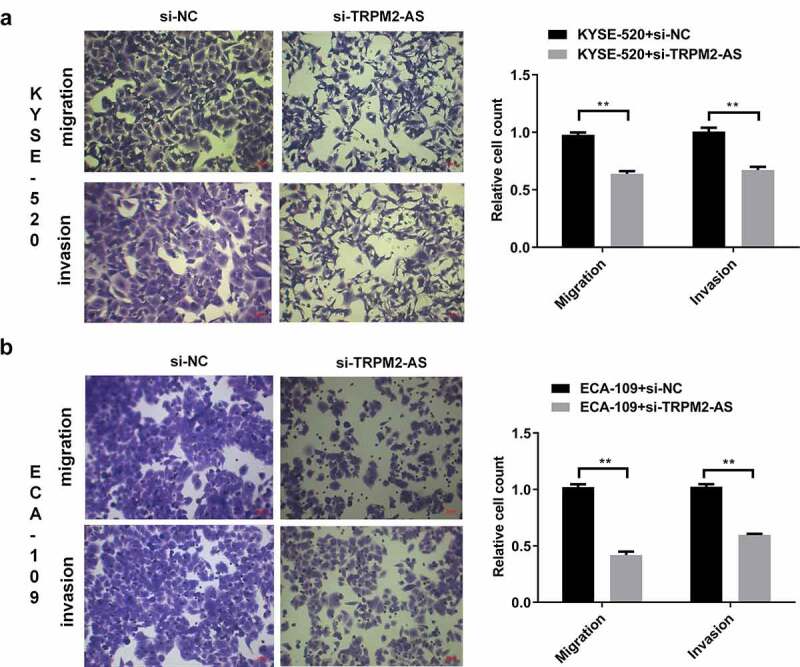
Silencing of lncRNA TRPM2-AS inhibits cell migration and invasion in esophageal cancer cells.

Metastasis is associated with cell invasion of nearby tissue and spreading to distant sites, contributing to cancer-related death [35]. We next determined the effects of lncRNA TRPM2-AS on cell migration and invasion by transwell assay. Silencing lncRNA TRPM2-AS significantly reduced cell migration and invasion in KYSE-520 cells by 34.8% and 33.3%, respectively ((Figure 4a), p < 0.05). We observed similar effects of si-TRPM2-AS on cell migration and invasion in ECA-109 cells (Figure 4b). These results suggest that lncRNA TRPM2-AS knockdown suppresses EC metastasis.

(A) Cell migration and invasion results of KYSE-520 cells determined at 48 h post cell seeding in the transwell chambers. (B) Cell migration and invasion results of ECA-109 cells determined at 48 h post cell seeding in the transwell chambers. **p < 0.01, n = 3.

### 3.4 LncRNA TRPM2-AS knockdown suppresses the epithelial–mesenchymal transitions (EMT) progress

**Figure 5.**
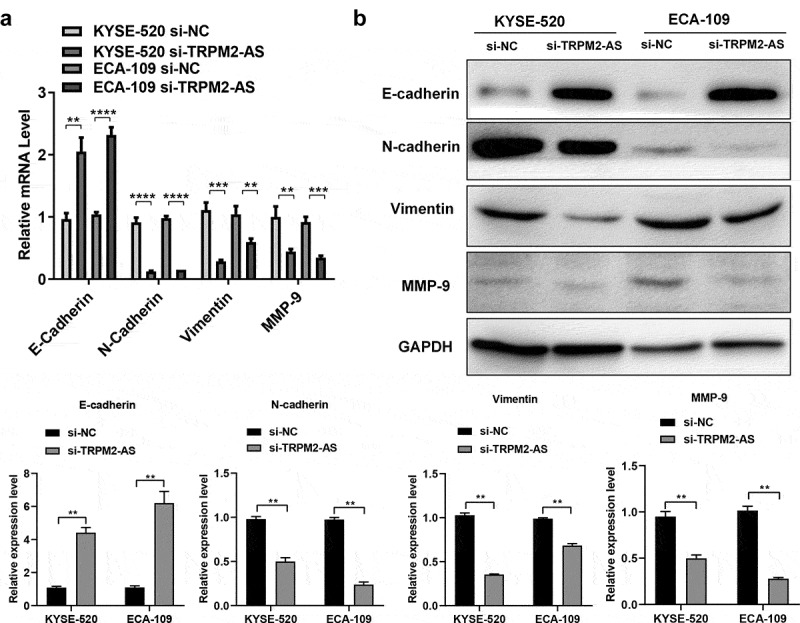
LncRNA TRPM2-AS knockdown suppresses the epithelial–mesenchymal transitions (EMT) progress in esophageal cancer cells.

EMT is essential for cancer cell migration, invasion, and apoptosis resistance, contributing to carcinogenesis [36]. We next determined EMT-related genes and proteins expression in EC cells after transfection of si-TRPM2-AS. LncTRPM2-AS silencing significantly inhibited neural cadherin (N-cadherin), Vimentin, and matrix metallopeptidase 9 (MMP-9) mRNA expressions while enhanced epithelial cadherin (E-cadherin) mRNA expression in both EC cell lines ((Figure 5a), p < 0.05). In line with gene expression results, Western blotting results showed similar effects of lncRNA TRPM2-AS knockdown on the protein expressions of these EMT markers (Figure 5b). Hence, these results demonstrate that lncRNA TRPM2-AS knockdown blunts the EMT progress in EC.

(A) EMT-related genes expressions were determined by quantitative real-time polymerase-chain reaction in KYSE-520 cells and ECA-109 cells after lncRNA TRPM2-AS knockdown. (B) Epithelial cadherin (E-cadherin), neural cadherin (N-cadherin), Vimentin, and matrix metallopeptidase 9 (MMP-9) protein expressions were determined by Western blotting in KYSE-520 and ECA-109 cells after lncRNA TRPM2-AS knockdown. *p < 0.05, **p < 0.01, ***p < 0.001, ****p < 0.0001, n = 3.

### 3.5 LncRNA TRPM2-AS regulates the expression of miR-1291, miR-6852-5p, and miR-138-5p in EC cells

**Figure 6.**
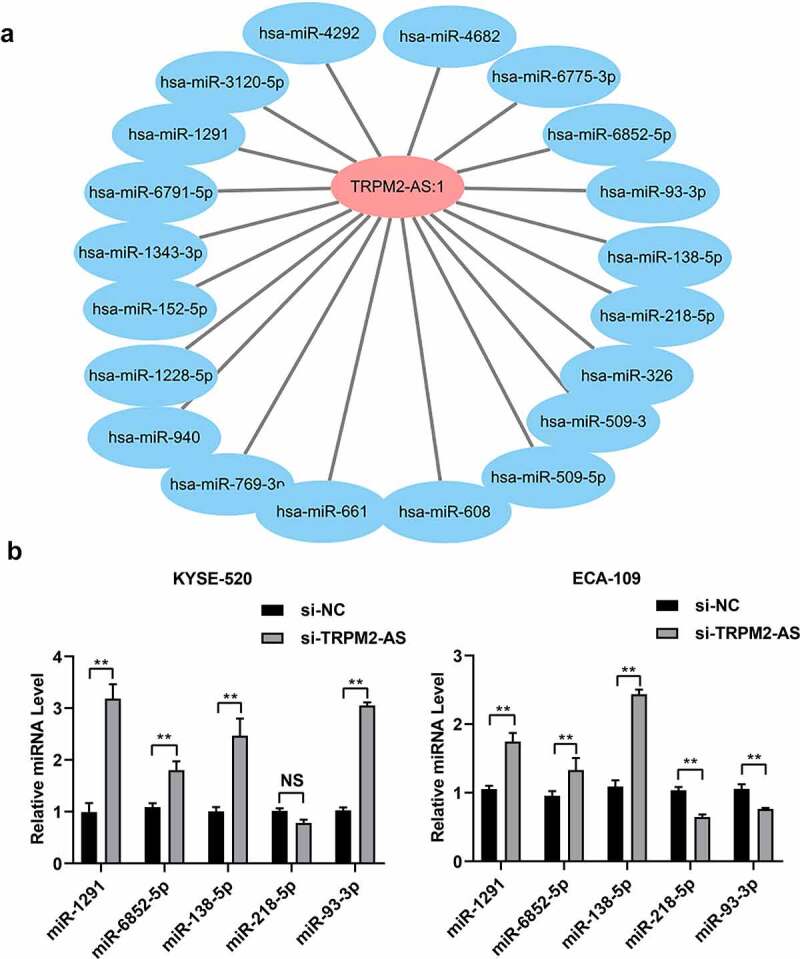
LncRNA TRPM2-AS regulates miR-1291, miR-6852-5p, and miR-138-5p expression in esophageal cancer cells.

We further explored the potential downstream targets of lncRNA TRPM2-AS in EC. We used miRnada and miTarbase to predict the potential miRNAs targeted by lncRNA TRPM2-AS and subsequently constructed the lncRNA TRPM2-AS-miRNAs interaction network using cytoscape. We found a total of 410 miRNAs potentially interacting with lncRNA TRPM2-AS (Supplementary table S1). (Figure 6a) showed the lncRNA TRPM2-AS-miRNA interaction network, including 20 miRNAs with top scores. Among them, the expressions of five miRNAs, i.e., miR-1291, miR-6852-5p, miR-138-5p, miR-218-5p, and miR-93-3p, were downregulated in several types of cancer, e.g., esophageal cancer, colorectal cancer, and hepatocellular cancer [37–41]. We then carried out qRT-PCR to validate their expression in EC cells. The results showed that si-TRPM2-AS significantly increased the expression of miR-1291, miR-6852-5p, and miR-138-5p in both KYSE-520 cells and ECA-109 cells (Figure 6b). In addition, miR-218-5p expression significantly decreased in lncTRPM2-AS knockdown ECA-109 cells compared with si-NC group, but no significant difference in KYSE-520 cells with lncTRPM2-AS knockdown group compared with si-NC group (Figure 6b). LncTRPM2-AS knockdown in the KYSE-520 cells enhanced miR-93-3p expression when compared with si-NC group; while lncTRPM2-AS knockdown reduced miR-93-3p expression in ECA-109 cells compared with si-NC group (Figure 6b). These results indicate that miR-1291, miR-6852-5p, and miR-138-5p might serve as the downstream molecules accounting for the cell biological functions of lncRNA TRPM2-AS.

(A) The interaction network between lncTRPM2-AS and potential targeting miRNAs was constructed using cytoscape. (B) The expression of miR-1291, miR-6852-5p, miR-138-5p, miR-218-5p, and miR-93-3p were determined using quantitative real-time polymerase-chain reaction in KYSE-520 cells and ECA-109 cells after lncRNA TRPM2-AS knockdown. NS: no significance; **p < 0.01, n = 3.

## 4. Discussion

Our study found that lncRNA TRPM2-AS was upregulated in the EC tissues and cell lines compared to respective control LncRNA TRPM2-AS silencing suppressed EC cell proliferation, migration, and invasion while promoted cell apoptosis. LncRNA TRPM2-AS knockdown also inhibited EMT progress. In addition, LncTRPM2-AS knockdown increased the expressions of miR-1291, miR-6852-5p, miR-138-5p.

LncRNA TRPM2-AS was found for the first time in prostate cancer, in which the lncRNA TRPM2-AS expression was upregulated and induced the progression of prostate cancer [31]. Subsequent investigations also showed that lncRNA TRPM2-AS was highly expressed in various types of cancer, and its high expression level indicates poor prognosis of cancers, e.g., non-small cell lung cancer [42], gastric cancer [18], ovarian cancer [21], and retinoblastoma [23]. LncRNA TRPM2-AS overexpression positively regulated cancer progression via promoting cancer cell proliferation, migration, invasion, and drug resistance, while suppressing cell apoptosis in many types of cancer [18,20–23,25–29,42]. In comparison, silencing of lncRNA TRPM2-AS showed opposite effects on cancer cell biological functions [18,19,42,43]. In line with these previous studies, our study showed that lncRNA TRPM2-AS expression increased in EC and silencing of TRPM2-AS inhibited cell proliferation, migration, and invasion, while enhanced cell apoptosis. It suggests that high expression of lncRNA TRPM2 is associated with poor prognosis of EC, which is worthy of future investigations.

EMT is a carcinogenic process that facilitate cancer metastasis in terms of promoting cell migration, invasion, and apoptosis resistance [36]. LncRNA DDX11 antisense RNA 1 promotes EMT in EC participating in cancer metastasis [44]. LncRNA TRPM2-AS also promoted EMT in laryngeal squamous cell carcinoma (LSCC) [28]. Consistently, our study showed that lncRNA TRPM2-AS silencing increased E-cadherin mRNA and protein expressions while reduced N-cadherin, vimentin, and MMP-9 mRNA and protein expressions in EC cell lines, which demonstrates that lncRNA TRPM2-AS positively regulates EMT process in EC.

A series of studies demonstrate that miRNAs are crucial downstream targets accounting for the effects of lncRNA TRPM2-AS on cancer cell growth and metastasis [21–23,27–29]. We found that lncRNA TRPM2-AS might target a total of 410 miRNAs predicted by public databases. Among these miRNAs with high scores, five miRNAs, including miR-1291, miR-6852-5p, miR-138-5p, miR-218-5p, and miR-93-3p, were previously shown to participate in cancer pathogenesis [26,45–48]. This study validated that only miR-1291, miR-6852-5p, and miR-138-5p were increased by lncTRPM2-AS knockdown. Among these three miRNAs, only miR-138-5p has published evidence that it was directly targeted by lncRNA TRPM2-AS and thus regulating downstream signaling, e.g., PI3K/AKT in non-small cell lung cancer, syndecan 3 (SDC3) in ovarian cancer, and urokinase in gastric adenocarcinoma [20,21,26]. MicroRNA-138 expression was associated with chemosensitivity and radiosensitivity of EC treatment [49,50]. Thus, we can speculate that miR-138-5p is a critical downstream target of lncRNA TRPM2 in EC, regulating EC cell growth and metastasis. MicroRNA-1291 was downregulated in ESCC, which promotes cell proliferation and invasion while inhibits cell apoptosis by regulating the expression of targets mucin 1 [37]. The role of miR-6852-5p in EC remains unknown. Although, our findings showed that the expressions of miR-1291, miR-6852-5p, and miR-138-5p could be regulated by lncRNA TRPM2-AS, the relationship between these three miRNAs in EC remains unclear. Future investigations are in need to illustrate whether lncRNA TRPM2-AS directly targets miR-1291 and miR-6852, and the roles of lncRNA TRPM2-AS-miRNAs interaction network in EC pathogenesis.

## 5. Conclusion

This study demonstrates for the first time that overexpression of lncRNA TRPM2-AS in EC promotes the growth and metastasis of EC likely through targeting miR-1291, miR-6852-5p, and miR-138-5p. Our findings provide potential novel therapeutic targets for EC treatment.

## Supplementary Material

Supplemental Material

## Data Availability

All Data are showed in this study.
